# Effectiveness of a training program for the acquisition of motor milestones in infants: a randomized clinical trial

**DOI:** 10.1186/s13052-025-01849-4

**Published:** 2025-01-31

**Authors:** Luis Fernández-Sola, Beatriz Cano-Díez, Yessica Pons-Solaz, Begoña Vera-Egido, Sergio Moreno-González

**Affiliations:** 1 Health Sciences Faculty, San Jorge University, 50830 Villanueva de Gállego, Saragossa, Spain; 2 Medicine Faculty, San Pablo CEU University, CEU Universities, 28660 Boadilla del Monte, Madrid, Spain; 3 Niddo Fisioterapia y Espacio de Crianza, 50001 Saragossa, Spain; 4 Nuestra Señora de Gracia Hospital, 50004 Saragossa, Spain

**Keywords:** Alberta Infant Motor Scale, Training program, Motor development, Motor milestones

## Abstract

**Background:**

In infants, the acquisition of all motor milestones is considered an expression of correct motor development during the first months of life. An association between typical motor development of the newborn and cognitive areas has been established. Few studies have evaluated the efficiency of parents’ knowledge of expected milestones in healthy infants.

This study aims to determine whether parents’ knowledge of specific tasks can improve the achievement of all gross motor milestones in the newborn.

**Method:**

The current study examined gross motor development in term-born infants without pathologies at 9, 12, and 15 months and the effectiveness of a training program developed for parents. The research group comprised 82 full-term infants divided into an experimental group (EG) and a control group (CG) of 41 subjects each. A randomized clinical trial study was performed. The routine follow-up program consisted of four informative sessions on the experimental group at the beginning of each trimester with information about the expected motor milestones and how to stimulate their infants to achieve them. The gross motor development of the participants was measured using the Alberta Infant Motor Scale. An ANCOVA test was performed to assess the possible influence of sex, type of birth, or the presence of siblings controlled and uncontrolled as confounding variables on the results.

**Results:**

The initial baseline assessment showed no statistical differences between groups (*p* > *0,05*). After controlling confounding variables, at 9 months the EG scored 5,5 points higher than the CG (*p* < *0,001*). At 12 months, EG scored 3,7 points higher than CG (*p* < *0,001*). At 15 months, EG scored 2,2 points higher than CG (*p* = *0,001*).

The experimental group scored significantly higher, with a 25-point higher percentile in each assessment.

**Conclusion:**

A learning program aimed at increasing parents’ knowledge of their infant´s gross motor development improved it. The information collected will help professionals who support parents in monitoring their babies.

Future studies using larger sample sizes, analysing other domains of global infant development, or investigating the possible influence of other parental factors are recommended.

**Trial registration:**

ClinicalTrials.gov ID NCT04693494. Registered December 28, 2020, retrospectively registered. https://clinicaltrials.gov/study/NCT04693494.

**Supplementary Information:**

The online version contains supplementary material available at 10.1186/s13052-025-01849-4.

## Article summary

Providing parents with accurate information about how to support their child's gross motor development in the first year of life can improve it. Motor milestones in a sample of 82 infants were measured with the Alberta Infant Motor Scale.

## What is known on this subject

Motor achievements are considered an expression of proper motor development and multiple studies have established their association with cognitive development. Staying in the prone position while awake is one of the American Academy of Pediatrics' recommendations for appropriate motor development.

## What the study adds

Parental knowledge of the motor milestones to be expected at each period and how to help infants achieve them, together with the reinforcement of known recommendations, help to improve gross motor development in the first year of life.

## Background

Healthcare professionals working in pediatrics with infants should be aware of typical motor development to determine if a particular individual has any variation from what is considered normal [1], understood as that which is most frequent, from a statistical perspective.

The human species has a completely immature central nervous system (CNS) at birth. The maturation of the cerebellum is considered critical for motor skills, but its development is equally important for cognitive skills in which the cortex is central [2]. Likewise, the acquisition of these cortically controlled human skills, such as reading and writing, requires complex systems of vestibular–ocular–manual coordination. In the motor area, the expression of this correct maturation is found in the successive acquisition of what is known as motor milestones: “natural motor behaviors that can be grasped with precision, which appear during the first months of life, with variation in the configuration, arrangement, and age at which they emerge” [3]. These motor milestones are the product of development, although the process followed to reach them is equally important [4, 5]. Examples of motor milestones include asymmetric support, rolling, creeping, sitting or crawling. Occasionally, in children without known disorders, not all motor milestones described in the literature are achieved. For their acquisition, the current theory of dynamic systems [6–8] highlights the importance, along with maturation, of the environment in which the newborn develops and the tasks (referring to the development that takes place through experience and learning) it faces [9, 10]. A lack of adequate stimuli is one of the conditioning factors that could prevent motor maturation from following the desired pathway [11].

Most of the literature on motor development shows that related research has focused on children with some pathology [12], who were born prematurely [13], or who had low birth weight [14], and existing interventions in health systems have shown to be effective, but few studies have sought to analyse the effect of stimulation in infants without alterations.

Multiple previous studies [11, 15–18] have established an association between typical motor development in the first months of life and cognitive areas. These include academic level [19], problems in reading [20], memory and processing speed [21], and language development [22–24].

The lack of studies analysing the effect of the environment and experiences on motor development in children without pathology and its relation to cognitive areas justifies the need for research. This study aimed to assess the gross motor development of a sample of infants at the end of their first year of life and to determine the effectiveness of a parent training program in positively influencing such development. This program includes information about the motor milestones that the infant is expected to reach at each point and what stimuli to offer to help the infant achieve them. It also seeks to evaluate not only a specific moment (12 months) but also successive measurements around the first year of life (9, 12, and 15 months) that confirm the data and provide information on the acquisition process of motor achievements. It is essential to achieve this objective by effectively transmitting relevant information to parents and improving their adherence to recommendations, using the strategies suggested by education specialists for improving knowledge learning [25–28].

In this context, the value of our findings in this field is expected to be high.

## Patients and Methods

### Study design

This research was designed with a randomized clinical trial model (ClinicalTrials.gov ID NCT04693494) to evaluate the effects of the independent variables (infant-parent training program) on the dependent variable (gross motor development of the infant). Since the aim was to examine the effectiveness of the routine parent training program for infants and to compare the results with those of infants who followed the check-ups provided by health services, this model was chosen by dividing the research sample into an experimental group (EG) and a control group (CG). The evaluators were masked, and the infants' spontaneous behavior assessed by the scale could not be conditioned by knowing to which group they belonged.

## Research population and sample

**Fig. 1:**
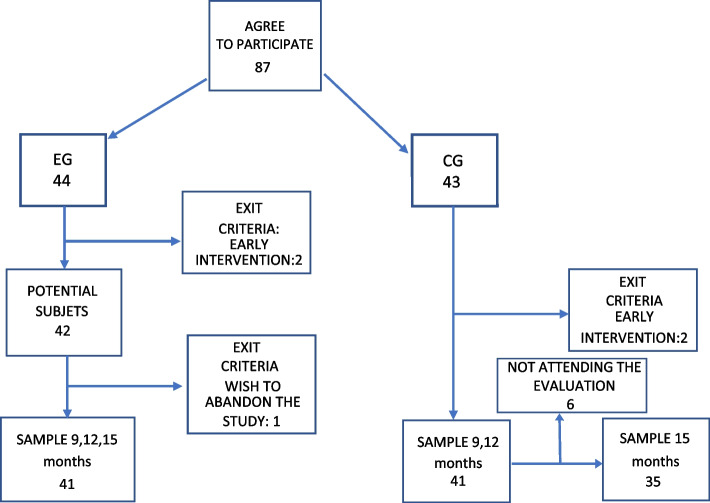
Participant flowchart

Recruitment took place in 2020. The flowchart of the participants is shown in Fig. 1. The sample size was calculated using the Epidat 3.1 software based on the required score to move from the 25th to the 50th and 50th to the 75th percentiles at 12 months in the original version of the Alberta Infant Motor Scale (AIMS), with a confidence level of 95%. The final investigation sample consisted of 82 infants without pathologies born at term in the Zaragoza II Health Sector, the sector with the highest number of births in the Community of Aragon (Spain). To form the participating groups, 87 infants were recruited by the main investigator at the Miguel Servet University Hospital in Zaragoza within hours following birth. All subjects were required to have a gestational age between 37 and 42 weeks, an Apgar score equal to or greater than 7 at one and five minutes after birth, a normal neurological examination and sensory tests in routine pediatric examination, no orthopedic alterations or known diseases, and no required hospital admission. Multiple births and those with orthopedic, genetic, or congenital pathology data were excluded. The randomization list was generated using a computer program (www.randomizer.org), with a masked person outside the study responsible for generating the list and assigning participants to groups. Excluded from the research were the infants in both groups whose parents wished to abandon the study as well as infants who required admission to the hospital from the time of inclusion, those who had an orthopedic injury, or those who required physiotherapy treatment or referral to the Early Intervention Service.

With these criteria, the exits recorded consisted of two cases in the EG and two in the CG for requiring a referral to the Early Intervention Service, and one case in the EG in which the parents indicated that they had not followed the guidelines for "personal motivations." For the 15-month assessment, six losses in the CG did not turn up for assessment when requested to do so.

### Information about the Group of Infants Studied

Information on the descriptive characteristics of the participating infants in both groups is presented in Table 1.

**Table 1:** Descriptive characteristics of the participating infants

*Variable*	*Global Sample*	*CG*	*EG*	*p*
	***n (%)***	***n (%)***	***n (%)***	
Sex				
Male	40 (48.8%)	14 (34.1%)	26 (63.4%)	0.015
Female	42 (51.2%)	27 (65.9%)	15 (36.6%)	
Type of delivery				
Cesarean section	13 (15.9%)	8 (19.5%)	5 (12.2%)	0.547
Vaginal	69 (84.1%)	33 (80.5%)	36 (87.8%)	
Order of birth				
First-born	46 (56.1%)	18 (43.9%)	28 (68.3%)	0.045
Not first-born	36 (43.9%)	23 (56.1%)	13 (31.7%)	

Analysis of the descriptive characteristics revealed that 48.8% of the infants were male, although the studied groups were not homogeneous, with 63.4% of the EG being male, a percentage that is slightly more than 29 points greater than that of the CG. Regarding the type of delivery, 19.5% of cesarean sections in the CG and 12.2% in the EG made up two homogeneous groups in terms of this variable. In addition, the number of first-born infants was recorded, and two nonhomogeneous groups were formed, with 68.3% of first-born infants in the EG and only 43.9% in the CG.

### Motor Assessment Scale

The gross motor development assessment tool used in this research, the AIMS [29], is an observational scale without any manual intervention that assesses 58 items divided into four subgroups: prone decubitus (21), supine decubitus (9), seated (12), and standing (16). Each of these items corresponds to a motor achievement, and the raw score obtained after adding up the observed milestones corresponds to a percentile that places the infant within the population of his or her age. This scale is characterized by high interobserver reliability and high concurrent validity with other scales [30].

Caution is advised by the authors in the interpretation of a low percentile score, as it has been observed that typically developing infants show fluctuations in their AIMS score from birth to autonomous walking [31]. Similarly, other authors [32] advise conducting evaluations in series rather than in isolation to draw more reliable conclusions.

### Learning Strategies

Previous research [33, 34] has suggested the importance of improving parental adherence to the recommendations offered and promoting access to information to improve infants' motor development. Jennings et al. [35] analysed the importance of how information is provided to new parents to achieve greater effectiveness in their education in terms of appropriate postures for their infants, with the combination of a visit by a professional (verbal information), together with an explanatory video and documentation with graphic information obtaining the best results in terms of parents following the guidelines set. The existing literature from education specialists [25–28] seems to point to this combination of learning strategies to improve knowledge acquisition.

### Process

In this research, the physiotherapists' routine interventions with the parents of the infants, consisting of four information sessions, were evaluated. The intervention consisted of face-to-face sessions, pamphlets, and videos of two YouTube channels at the beginning of each trimester. The design of these sessions and their content were shaped by an in-depth review of the literature on the most effective educational strategies for the transmission of knowledge, the motor milestones that the infant is expected to acquire in the first months of life [36], and the recommendations of the American Academy of Pediatrics (AAP) on proper infant positions [37], supported by various research studies [33].

In the first phase of the design, we decided how the information would be transmitted. This consisted of a face-to-face briefing session of approximately 30–40 min at the beginning of each trimester of the infant's first year of life; in addition, at the end of each session, the parents were provided a pamphlet with the main points explained in the session. Finally, they were provided a link to two YouTube channels [38, 39] produced by physiotherapists, the content of which had been previously reviewed by the researchers, emphasizing advice and strategies through explanatory videos.

To encourage adherence to the intervention, one month after each session, an email was sent to the EG parents to recall the key objectives for the period in which their infant was and the basic recommendations for achieving them. The researcher's email address was provided so that parents could contact him with any doubts that might arise regarding the information provided.

In the second step, the information to be conveyed in each session was selected. The content of these consisted of basic stimulation tips, expected postures, and sensory experiences as part of interactions with caregivers, as summarized in Table 2.

**Table 2:** Content of the briefing sessions

*Session*	*Content*
1st session (0–6 weeks old)	- Position the infant in the prone position, awake and monitored, for at least four periods of five minutes per day, and increase this time progressively according to the infant's tolerance [37, 40]. - Carry the infant in an ergonomic babywearing system for at least one hour a day [41–43]. - Frequently touch the infant's skin, especially their hands, feet, and mouth, and engage in periods of "skin-to-skin" when possible [44–47].
2nd session (3 months old)	- Increase the time spent in the prone position, making it the reference position when the infant is awake, and maintain ergonomic babywearing for a minimum of 1 h/day. - Progress onto asymmetrical support: In the prone position, offer the child toys on both sides, seeking dissociation of the limbs [36]. - Progress toward turning from supine to prone: In supine, start by offering toys on both sides and evolve to offering them in the midline, in front of their eyes [36, 48, 49]. - Increase the infant's movement while holding him or her in your arms staring at the parents, without making any sudden movements [50].
3rd session (6 months old)	- Retain prone as the reference position for play until he or she starts to crawl. - Progress to reaching for objects placed above and turning from prone to supine; in the prone position, offer toys higher and higher and on both sides [36].
4th session (9 months old)	- Place toys far away, encouraging movement by crawling or creeping [36]. - When movement is fluid, place toys on a low piece of furniture to stimulate the onset of standing upright. - Correctly progress from the initial standing position (the infant is put in this position by pulling on the household furniture with his hands) to autonomous walking with its stages (sideways walking holding on to a piece of furniture, standing with trunk rotation, making the transition from one piece of furniture to the next), avoiding "helping him/her to walk" by holding his or her hands [36].

**Fig. 2:**
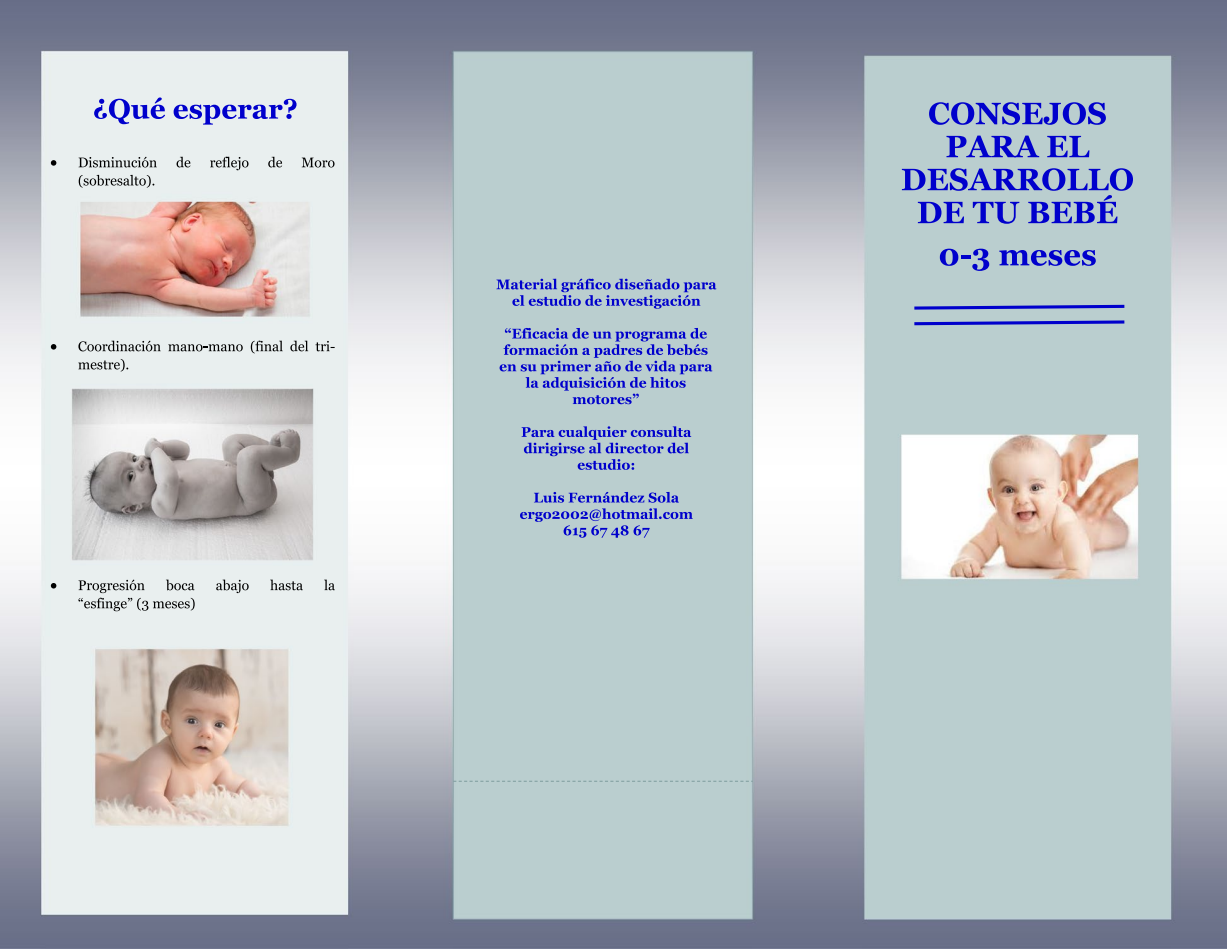
Graphic documents (pamphlet) provided

The graphic documents provided are shown in Fig. 2.

While the training program was being carried out, the CG was not subjected to any process other than their routine checkups in primary care pediatric services. The benefit of participating in the CG was the free additional gross motor development assessment performed. In addition, a complete assessment involving gross and fine motor development and social and speech areas, using validated tools, as well as advice for improving development, were offered to the participants in case of better results from the EG.

### Variables, data collection, and measuring instruments

The parents completed a questionnaire in which, in addition to the data concerning the type of delivery, weeks of gestation, or presence of siblings, they were required to reflect on the daily times they positioned the infant in the prone position and the times they carried the infant in a babywearing system from the age of two to eight months. They also indicated whether they had generally followed the guidelines set out in the training sessions. The purpose of the questionnaire was to monitor the adherence of the parents to the recommendations provided.

The variable measured and compared between the two groups was the total score obtained by administering the AIMS at 9, 12, and 15 months of age for both the EG and CG. The evaluation sheets of the equally validated Spanish version of the AIMS [51] were used. An initial assessment of the baseline data was carried out at 2 months of age, and the results are shown in Table 3.

**Table 3:** AIMS2 baseline data

*AIMS2*					
*Control of *_*Variables*_	***Control Group***	***Experimental Group***	***Mean Difference***	^***F(p)***^	***Partial eta square***
3 variables	7.99 [7.57; 8.41] ***23***	8.08 [7.66; 8.50] ***23***	0.091	F1,71 = 0.085 (0.771)	0.001
uncontrolled	8.00 [7.60; 8.40]	8.07 [7.68; 8.47]	0.073	F1,74 = 0.067 (0.796)	0.001

Mean [I.C. mean 95%] ***Percentile on the original scale***

^1^ ANCOVA

Since this scale is used to evaluate the spontaneous attitudes of the infant, it could be the case that at the time of the assessment, some circumstances would prevent a behavior similar to that which they would have demonstrated under optimal conditions. Therefore, if this happened, the parents were asked to film a video at home, when the infant was calm, in different assessment postures. This option is feasible for parents of children with typical motor development and has been validated in other research [52]. Six parents in the EG and seven in the CG were required to record home videos at the 15-month assessment.

The data collection during the evaluations was performed by three physiotherapy graduates trained in the administration of the AIMS. The assessment carried out to obtain the data followed the criterion of blinding the assessor, who did not know to which group the assessed infants belonged. To support the high interobserver reproducibility demonstrated by the scale, two meetings were held between the principal investigator and the three evaluators to standardize the scoring criteria and administration guidelines. These evaluations were carried out in an open-plan room that allowed the infants to move freely, and the three evaluators used the same objects and toys.

SPSS software version 25 (SPSS Inc. by IBM, Chicago, Il. USA) was used for computer processing of the data. To determine whether the groups were homogeneous in terms of sex, type of birth, and presence of older siblings, a chi-square test was carried out. To establish comparisons between the motor development of the two groups measured with the AIMS, after determining the normal distribution of this variable, an ANCOVA test was performed to assess the possible influence of sex, type of birth, or the presence of siblings controlled and uncontrolled as confounding variables on the results. The confidence interval was calculated at a confidence level of 95% (statistically significant at *p* < 0.05). The effect size was also calculated using partial eta-squared, and values above 0.14 were considered to indicate a high effect size.

### Ethical considerations

This research was approved by the Regional Research Ethics Committee (CEICA) and was authorized by Miguel Servet Hospital as a recruitment center. The participating parents signed an informed consent form, and the data and videos collected were processed following data protection legislation. The evaluators signed an affidavit agreeing not to disclose the participants’ data. All procedures followed the ethical principles for medical research involving human subjects as laid out in the Declaration of Helsinki.

## Results

The results of the initial baseline assessment are shown in Table 3. No statistical differences appeared with the confounding variables controlled and uncontrolled (*p* > *0,05*).

To determine whether the levels of gross motor development in the EG were positively affected by the training received by their parents, the results were analysed, and an ANCOVA test was used to determine whether the differences found were statistically significant. The effect size was also calculated for clinical significance.

Table 4 shows the data obtained at 9 months (AIMS9), 12 months (AIMS12), and 15 months (AIMS15) in the comparison of groups, with the three variables mentioned in Table 1 (sex, type of delivery, and presence of older siblings) controlled and uncontrolled.

**Table 4:** AIMS9, AIMS12 and AIMS15 data

***AIMS9***					
***Control of Variables***	***Control Group***	***Experimental Group***	***Mean Difference***	***F (p)***	***Partial eta Square***
3 variables	39.08 [37.10; 41.05] ***19***	44.61 [42.63; 46.58] ***45***	5.532	F1,77 = 14.451(< 0.001)	0.158
uncontrolled	39.44 [37.56; 41.32]	44.24 [42.37; 46.12]	4.805	F1,80 = 12.959 (0.001)	0.139
***AIMS12***					
***Control of Variables***	***Control Group***	***Experimental Group***	***Mean Difference***	***F (p)***	***Partial eta Square***
3 variables	49.38 [48.24; 50.51] ***11***	53.11 [51.98; 54.25] ***36***	3.737	F1,77 =19.898 (<0.001)	0.205
uncontrolled	49.59 [48.49; 50.68]	52.90 [51.81;54.00]	3.317	F1,80 =18.198 (0.001)	0.185
***AIMS15***					
***Control of Variables***	***Control Group***	***Experimental Group***	***Mean Difference***	***F (p)***	***Partial eta square***
3 variables	54.88 [53.99; 55.77] *<<5*	57.13 [56.31; 57.94] ***20***	2.247	F1,71 = 12.619(0.001)	0.151
uncontrolled	55.11 [54.27; 55.96]	56.93 [56.15; 57.71]	1.813	F1,74 = 9.844 (0.002)	0.117

Mean [I.C. mean 95%] ***Percentile on the original scale***

^1^ANCOVA

In the three assessments performed without controlling for confounding variables, statistically significant differences in AIMS gross motor development scores were obtained in favor of the EG. In the AIMS9, EG scored 4,8 points higher than CG (*p* = *0,001*). In the AIMS12, EG scored 3,3 points higher than CG (*p* = *0,001*). In the AIMS15, EG scored 1,8 points higher than CG (*p* = *0,002*).

When ANCOVA was performed to control for these possible confounding variables, these statistically significant differences in favour of the EG again appeared, so it can be affirmed that these variables did not influence the results obtained. In the AIMS9, EG scored 5,5 points higher than CG (*p* < *0,001*). In the AIMS12, EG scored 3,7 points higher than CG (*p* < *0,001*). In the AIMS15, EG scored 2,2 points higher than CG (*p* = *0,001*). In all the cases, the clinical significance expressed by the effect size (partial eta-squared test) confirmed the relevance of these differences.

## Discussion

An examination of the mean scores of both participant groups revealed that the EG, whose parents had received the training program, was superior in gross motor performance in all assessments performed, with this difference being statistically and clinically significant. The fact that the best results were maintained over three evaluations, with six months elapsed from the first to the third, allows us to affirm that this motor advantage did not occur in an isolated moment in time but that the process of motor development of the EG was better, demonstrating a greater acquisition of motor milestones at earlier times and in a sustained manner over time. These results are in line with those obtained by Jennings et al. [35] in their research offering parents a program similar to that used in the present study. The need to provide information to parents regarding optimal positions for their infants was also considered in research by Koren et al. [53] years ago and more recently by Orlando et al. [54]

The training that has been shown to be beneficial in obtaining these results should focus especially on the recommendations widely put forward by the AAP [37] and included in the literature [40]. This training includes the need to place infants in the prone position for several periods a day while awake and under supervision.

Given these results, the sex of the infant, the type of delivery, and the presence of older siblings do not seem to influence the acquisition of gross motor milestones. Similar conclusions regarding gender were reached by Piper and Darrah [29] with the sample used for the original scale, Pereira et al., [11] Flensborg-Madsen and Mortensen [55], and Ertem et al. [56] Only Morag [57] reported that male sex was a risk factor in his research. Regarding the type of delivery, Zhu et al. [58] and Obican et al. [59] also reported no relationship with motor development. Morag’s research [57] found cesarean delivery to be a risk factor. Rodrigues and Silva [60] found that children born by cesarean delivery scored lower in the locomotor skill domain, but still within the normal parameters for their age. However, Zaigham et al. [61] reported that infants delivered by prelabour cesarean section had significantly lower scores at 4-month evaluation and these differences remained at the 12-month assessment. Finally, few studies have analysed the possible influence of the presence of older siblings, with Leonard and Hill's study [62] also finding no influence of this variable.

The results of this study highlight the need to update educational materials for parents and to develop parenting education programs that focus on activities that promote early development. Placing babies in the prone position and knowing the expected milestones at each stage will help achieve this.

## Conclusions and Recommendations

Expanding the information provided by health services through programs that teach parents what milestones to expect in the first year of life and how to stimulate their acquisition is beneficial for gross motor performance. Among the most relevant stimuli is positioning the infants in a prone position and monitoring them frequently when awake. A combination of strategies such as oral transmission, graphic documents, and audiovisual content is effective in transmitting the intended knowledge.

## Limitations and Implications

The results of this research showed that considering the possible future repercussions of atypical gross motor development, training programs should be delivered in addition to the insufficient amount of information received from primary care services.

As the results of this study were obtained only with infants in one health sector in one region of Spain, there are limitations to the generalizability of the conclusions drawn.

Similarly, the scale used was exclusively quantitative, referring to gross motor development, and did not consider the maturity or fluency with which different motor milestones are achieved or other areas of development, such as fine motor performance and language, or developments in the social sphere.

To support these results, future studies that include different samples and larger sample sizes, as well as the use of other validated scales analysing other domains of global infant development, are recommended.

Similarly, the possible influence of parental factors, such as socioeconomic status, educational level, or maternal age, is proposed as a future line of research. Finally, it would be interesting to assess possible difficulties in other areas at school age among the participating children and whether these difficulties are linked to the results obtained here.

## Supplementary Information

Supplementary Material 1.

## Data Availability

The datasets used and/or analysed in the current study are available from the corresponding author upon reasonable request.

## Abbreviations

CNS: Central Nervous System
EG: Experimental group
CG: Control group
AIMS: Alberta Infant Motor Scale
AAP: American Academy of Pediatrics
