# Supplementary material for: Effectiveness of a training program for the acquisition of motor milestones in infants: a randomized clinical trial
Source: Ital J Pediatr. 2025 Jan 31;51:23. doi: 10.1186/s13052-025-01849-4 (PMC11786511; doi:10.1186/s13052-025-01849-4)

¿Qué esperar?

- Disminución de reflejo de Moro (sobresalto).

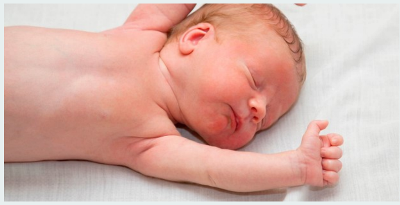

- Coordinación mano-mano (final del tri-mestre).

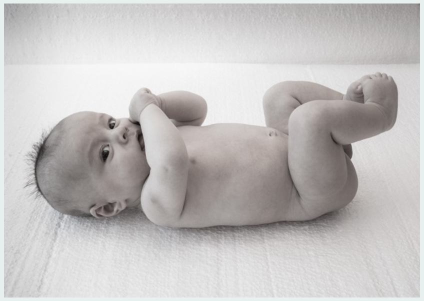

- Progresión boca abajo hasta la "esfinge" (3 meses)

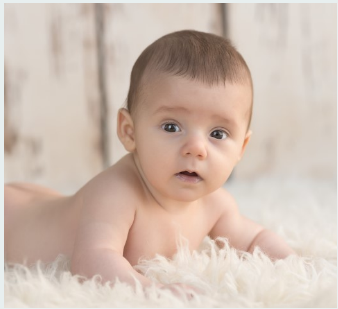

Material gráfico diseñado para el estudio de investigación

"Eficacia de un programa de formación a padres de bebés en su primer año de vida para la adquisición de hitos motores"

Para cualquier consulta dirigirse al director del estudio:

Luis Fernández Sola
ergo2002@hotmail.com
615 67 48 67

CONSEJOS PARA EL DESARROLLO DE TU BEBÉ

0-3 meses

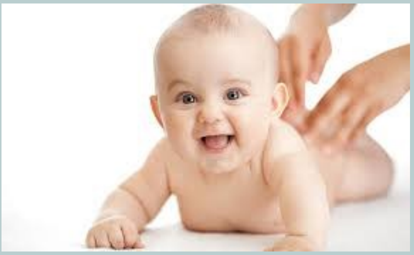

¿Qué hacer?

TACTO: una de las principales fuentes de información para su cerebro en esta etapa es la piel. Es fundamental TOCAR MUCHO, especialmente las manos, los pies y la boca.

Intenta pasar varios minutos al día PIEL CON PIEL.

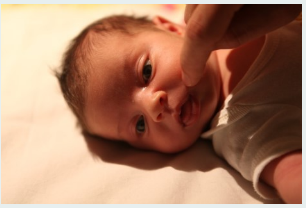

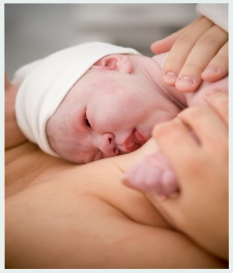

PORTEO: otra gran fuente de información es su sistema vestibular. Se encuentra en el oído y se activa con el MOVIMIENTO. El porteo debe ser un modo de transporte preferente. Debes caminar al menos 1h/día con tu bebé porteado. Existen sistemas adecuados a la edad del bebé y a las características de los padres. Pregunta a un especialista en porteo.

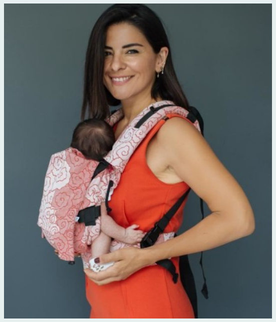

¿Qué hacer?

PRONO: recuerda que el desarrollo del bebé se realiza empujando el suelo. Desde que nace, debe pasar muchos momentos tumbado boca abajo DESPIERTO Y VIGILADO.

Recién nacido 5 minutos-5 veces/día y se va aumentando progresivamente.

Recuerda: dormido, boca arriba. Despierto, boca abajo.

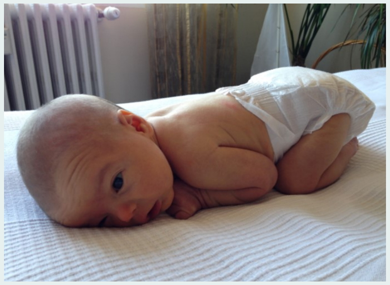

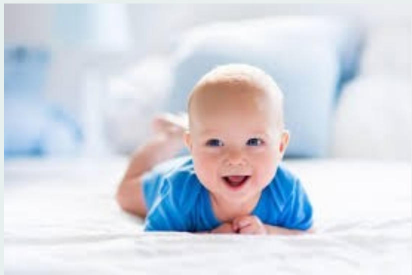

VERIFICA QUE...

- Es capaz de girar el cuello libremente a AMBOS LADOS.

¿Qué evitar?

- Los movimientos bruscos....
- ... y la falta de movimiento!
- Largos tiempos boca arriba y sin movimiento.

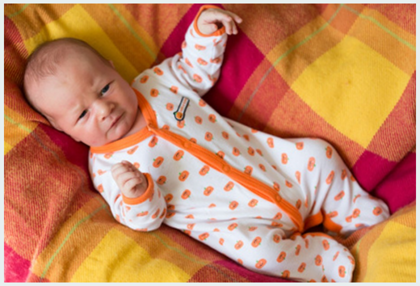

¿Qué esperar?

- Fijación de la mirada (6 semanas)

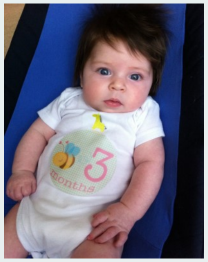

- Seguimiento hacia ambos lados y “posición de esgrima” (desde la 7ª semana)

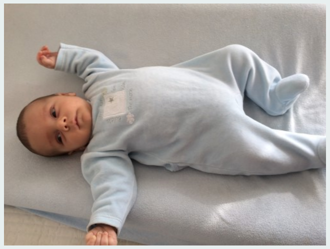

¿Qué esperar?

- Volteo de boca arriba a boca abajo.

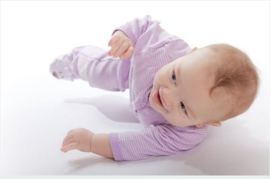

- Manos a pies con 6 meses

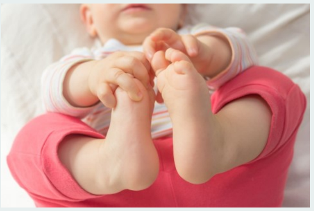

- Boca abajo brazos estirados estable con 6 meses

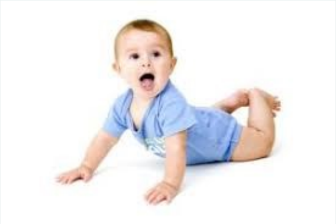

- Expresividad e imitación

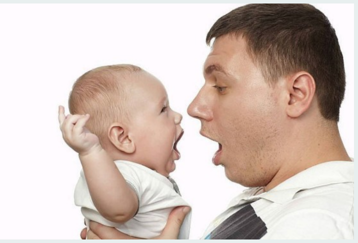

Material gráfico diseñado para el estudio de investigación

"Eficacia de un programa de formación a padres de bebés en su primer año de vida para la adquisición de hitos motores"

Para cualquier consulta dirigirse al director del estudio:

Luis Fernández Sola
ergo2002@hotmail.com
615 67 48 67

CONSEJOS PARA EL DESARROLLO DE TU BEBÉ

3-6 meses

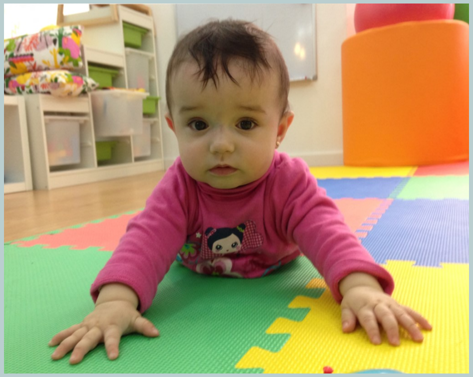

¿Qué hacer?

PORTEO Y MOVIMIENTO: portearlo siempre en VERTICAL sigue siendo fundamental. Y debemos ir aumentando el estímulo de movimiento que le ofrecemos.

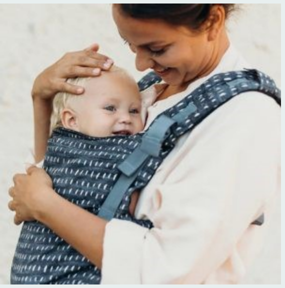

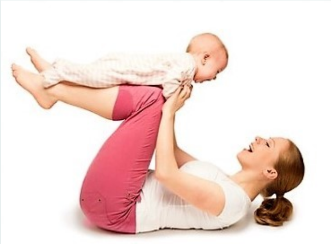

JUGAR BOCA ABAJO: debe ser la posición de preferencia. Ofrecemos juguetes a ambos lados y a la vez acariciamos la espalda.

Le balanceamos el cuerpo hacia ambos lados.

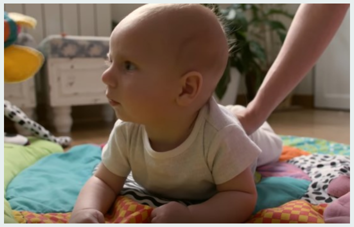

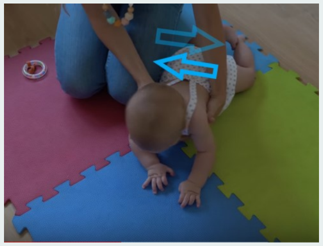

¿Qué hacer?

BOCA ABAJO: le damos soporte para que pueda sacar la mano doblando la rodilla del mismo lado y fijando el brazo contrario.

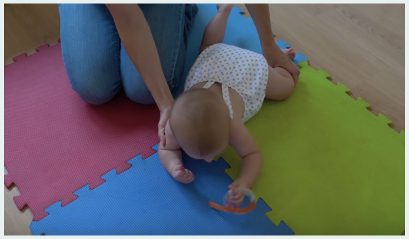

BOCA ARRIBA: ofrecemos a ambos lados objetos livianos y que pueda coger y llevarse a la boca. Llévaselos hacia el centro progresivamente. (4,5-5 meses) y pónselos lejos ayudándole a voltear.

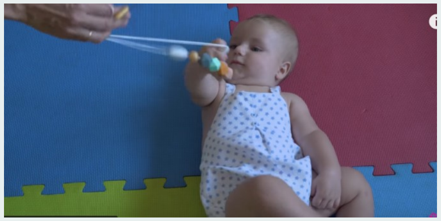

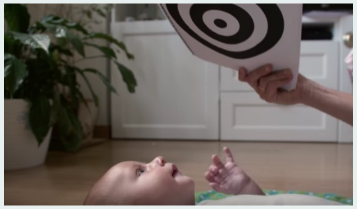

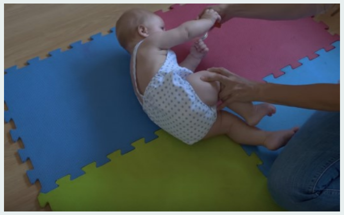

¿Qué evitar?

- Ponerle de pie
- Juegos móviles en el centro que no pueda agarrar

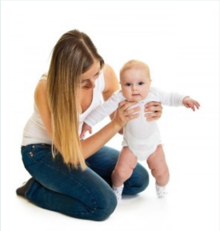

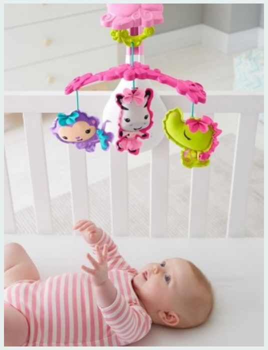

¿Qué esperar?

- Progresión en el apoyo asimétrico boca abajo

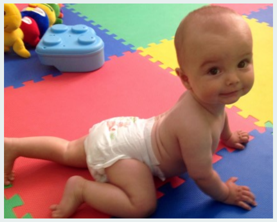

- Alcance de objetos boca arriba en el medio

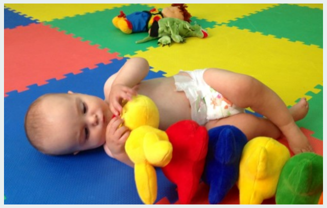

¿Qué esperar?

- Pinza radial y transferencia de juguetes

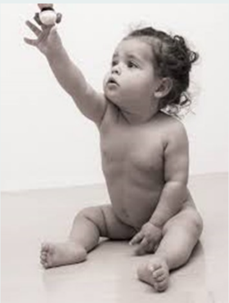
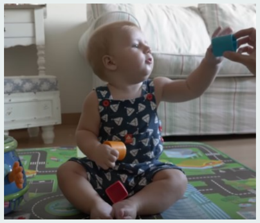

- Decúbito lateral estable y sedestación oblicua

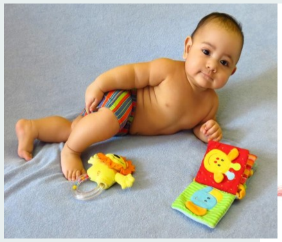
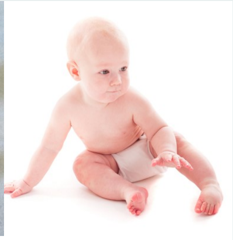

- Ansia social. Emociones. Sonidos.

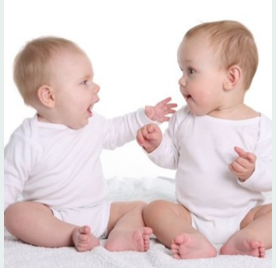

Material gráfico diseñado para el estudio de investigación

"Eficacia de un programa de formación a padres de bebés en su primer año de vida para la adquisición de hitos motores"

Para cualquier consulta dirigirse al director del estudio:

Luis Fernández Sola
ergo2002@hotmail.com
615 67 48 67

CONSEJOS PARA EL DESARROLLO DE TU BEBÉ

6-9 meses

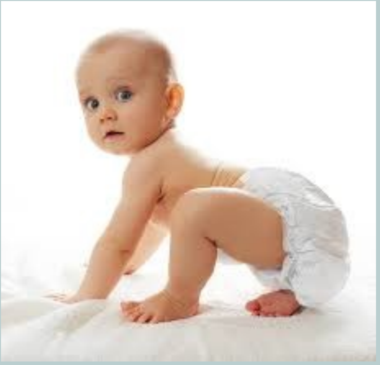

¿Qué hacer?

PORTEO Y MOVIMIENTO: portearlo sigue siendo importante, aunque menos SI SE HA HECHO CON ANTERIORIDAD. Y debemos ir aumentando el estímulo de movimiento que le ofrecemos.

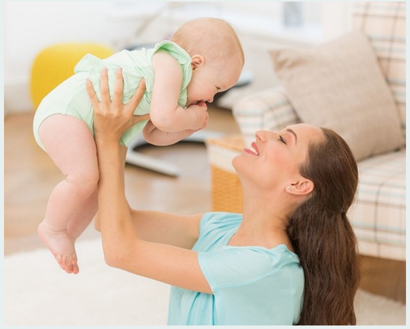

SIEMPRE POR LOS LADOS: deben darse los juguetes por ambos lados, buscando progresar en altura. El niño descubre el "ESPACIO DE ARRIBA"

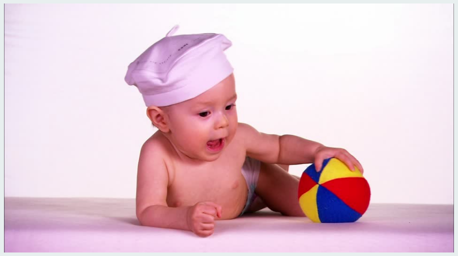

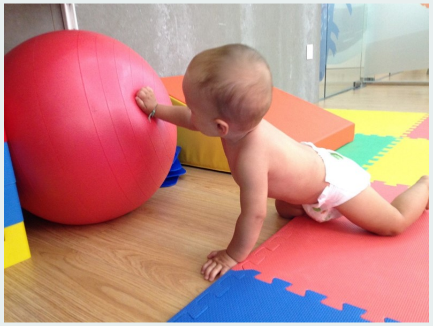

¿Qué hacer?

DESDE SENTADO: estímulos laterales para favorecer salida lateral

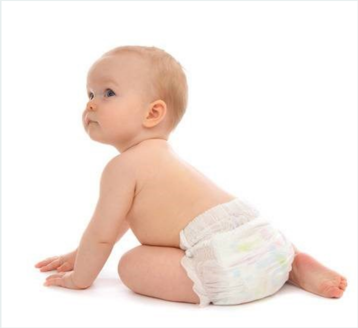

POTENCIAR DESPLAZAMIENTOS: poner juguetes lejos y cada vez más altos

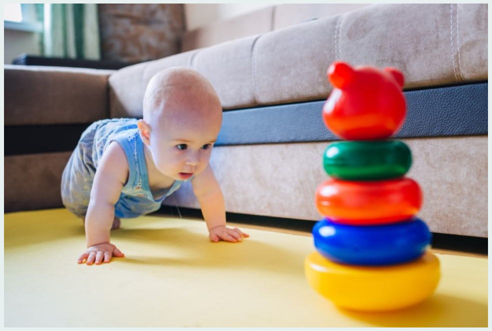

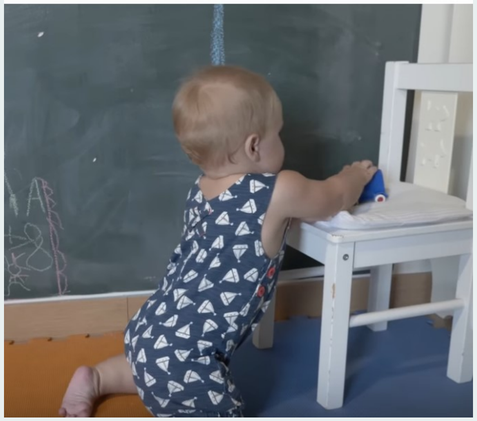

¿Qué evitar?

- Ponerle de pie

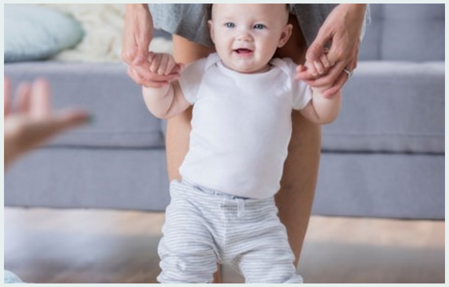

¿Qué esperar?

- Se levanta a apoyarse sobre sus rodillas. Desde ahí caerá lateralmente a sentado

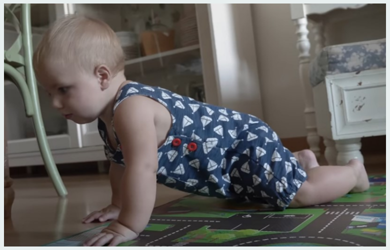

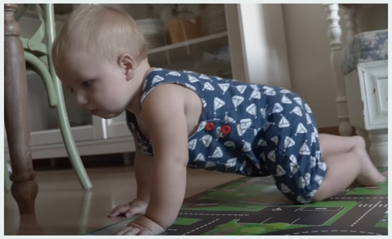

¿Qué evitar?

- "W sitting": especial atención en los primeros años de vida.

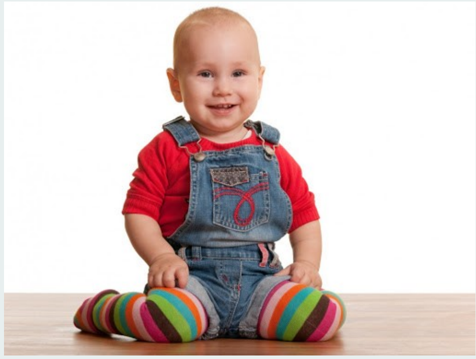

¿Qué esperar?

- Primeros pasos

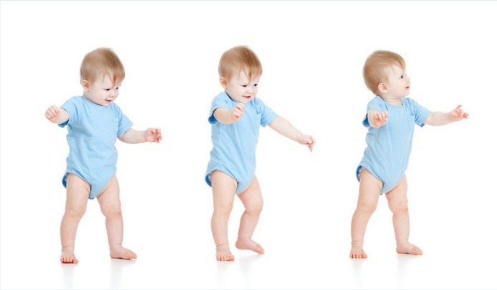

Material gráfico diseñado para el estudio de investigación

"Eficacia de un programa de formación a padres de bebés en su primer año de vida para la adquisición de hitos motores"

Para cualquier consulta dirigirse al director del estudio:

Luis Fernández Sola
ergo2002@hotmail.com
615 67 48 67

CONSEJOS PARA EL DESARROLLO DE TU BEBÉ

9-12 meses

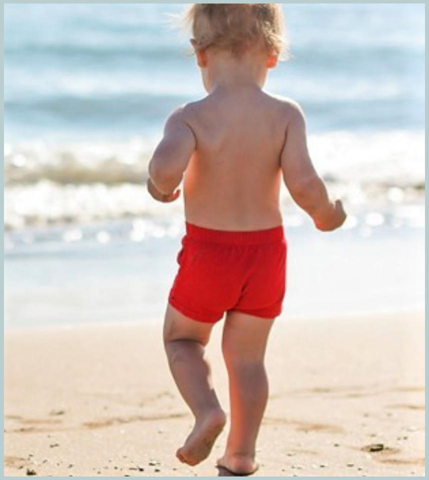

¿Qué hacer?

INTENSO DESEO DE ALCANZAR COSAS:

- Permitir la frustración de los fracasos

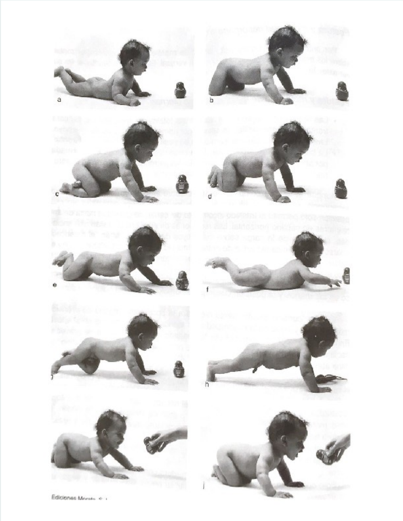

SIEMPRE POR LOS LADOS:

- Las transiciones sentado-gateo-sentado deben ser por los lados. Poner los juguetes lateralmente.

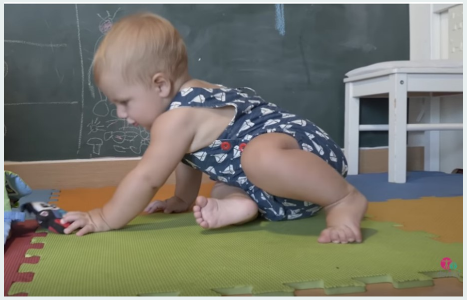

¿Qué hacer?

POTENCIAR DESPLAZAMIENTOS LATERALES CUANDO ESTÉ DE PIE

- Alejarle los juguetes lateralmente

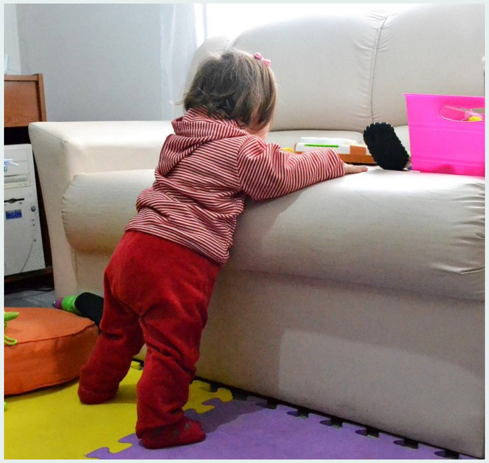

POTENCIAR DESPLAZAMIENTOS ENTRE MUEBLES:

- Poner juguetes en muebles contiguos: "marcha entre islas"

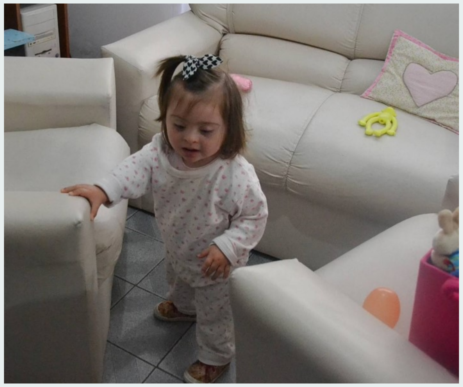

¿Qué hacer?

- Ayudar en transiciones de pie-cuclillas-de pie.

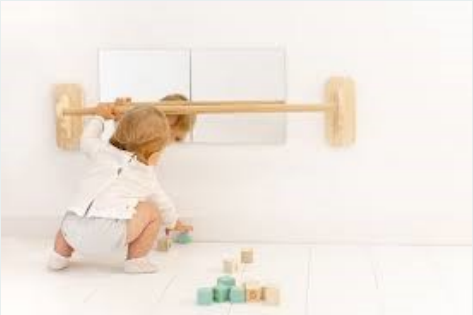

¿Qué evitar?

- Ninguna prisa por ayudarle a andar. Al menos, hasta que no haga con fluidez la "marcha entre islas"

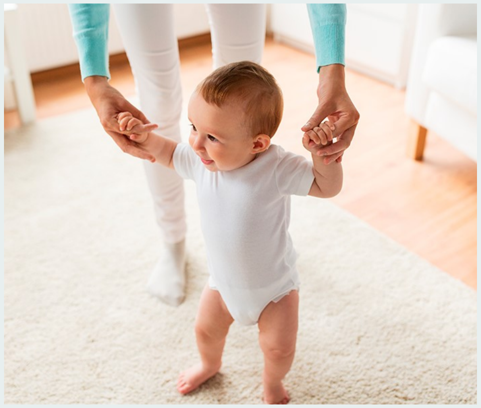

Supplement: Supplementary file 1 — Supplementary Material 1. [file 13052_2025_1849_MOESM1_ESM.pdf]
